# Traveling Thrombus in the Right Atrium: Is It the Final Destination?

**DOI:** 10.1155/2012/378282

**Published:** 2012-08-09

**Authors:** Maneesh Bhargava, Erhan Dincer

**Affiliations:** Division of Pulmonary, Allergy, Critical Care and Sleep Medicine, University of Minnesota, MN 55455, USA

## Abstract

Right heart thrombus is rare in structurally normal heart. Here, we report a 74-year-old man with a right atrial thrombus who presented with shortness of breath.

## 1. Introduction

Right heart thrombus in the absence of structural heart disease, atrial fibrillation, or catheter located in the heart is rare and usually represents a traveling clot from the venous system to the lung, known as right heart thrombi-in-transit (RHThIT). The optimal therapy for RHThIT remains controversial. We elected surgical thromboembolectomy for our patient. However, the thrombus in the right atrium had migrated to the pulmonary circulation during the surgery.

## 2. Case Report 

**Figure 1:**
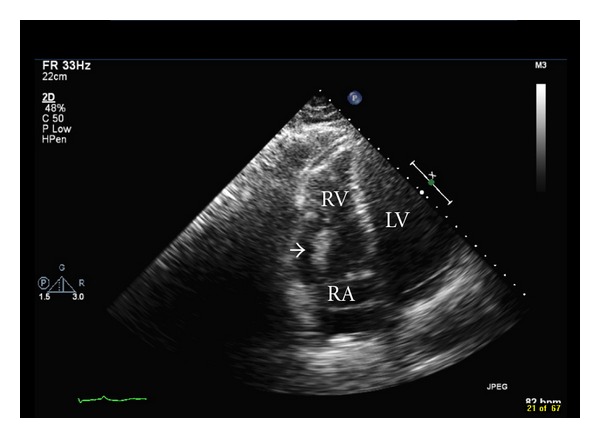
Transthoracic echocardiography showing a large right atrial thrombus (white arrow) extending into the right ventricle.

A 74-year-old man presented to our ER with shortness of breath. He had a recent history of air and car travel lasting seven hours. His past medical history was remarkable for DVT, prostate cancer, obstructive sleep apnea, hypertension, and chronic kidney disease stage 1. His examination was unremarkable except for mild tachycardia and hypoxemia at rest. Laboratory studies showed an elevated D-dimer, troponin, and BNP. An occlusive thrombus extending from the mid-thigh to the mid-calf on the right was seen on a Doppler study. A CT angiogram of the chest was not performed due to high creatinine but ventilation-perfusion scan showed a high probability for pulmonary embolism. Trans-thoracic echocardiography (TTE) revealed a large mobile mass extending from the right atrium through the tricuspid valve into the right ventricle (Figure 1). Right ventricle was mildly dilated with decreased systolic function.

**Figure 2:**
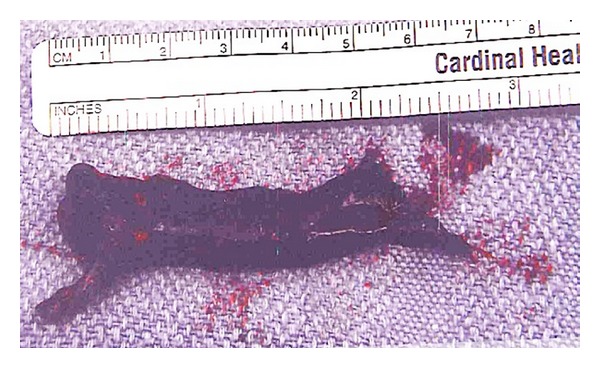
The thrombus removed from the left pulmonary artery.

A retrievable IVC Tulip filter was placed. After consultation with cardiology, pulmonary medicine, and cardiovascular surgery, it was decided to pursue surgical exploration with thrombectomy. The presence of the right atrial mass was confirmed by TTE prior to surgery. Right atrial exploration demonstrated no masses. A 2 cm incision was made in the pulmonary artery and a large thrombus was removed from the orifice of the left pulmonary artery (Figure 2). Pathologic examination showed laminated thrombus confirming diagnosis of pulmonary embolism.

Postoperatively in the ICU, the patient needed cardio-pulmonary resuscitation for pulseless electrical activity on two separate occasions within hours of surgery. Next four days, he required high degree of supportive care with pressors, ionotropes, mechanical ventilation, and inhaled epoprostenol. A repeat TTE showed severely decreased right ventricular systolic function. A CT scan of the chest showed extensive bilateral pulmonary emboli. The patient was liberated from mechanical ventilator on postoperative day eight. Thrombophilia panel was positive for lupus anticoagulant. He was discharged home on hospital day seventeen with a plan of indefinite anticoagulation.

## 3. Discussion

Right heart thrombus may develop within the right heart chambers (Type B) or they may be peripheral venous clots that accidentally lodge in the right heart on their way to the lungs (Type A), known as right heart thrombi-in-transit (RHThIT). RHThIT is a rare phenomenon in the absence of structural heart disease, atrial fibrillation, or a device located in the superior vena cava or the heart chambers, such as catheter or pacemaker leads. But, it can be seen in patients with thrombophilia, malignant tumors, Crohn's disease, and Behcet's disease.

Clinical consequences of RHThIT depend upon the clot size and overall clot burden. Sudden cardiovascular collapse is the worst outcome if the clot compromises the circulation in the heart or the pulmonary circulation. Therefore, RHThIT is considered to be an extreme therapeutic emergency [1, 2]. Case series reported high in-hospital mortality of 44.7% due to sudden pulmonary embolism [3]. The overall mortality rate in patients with RHThIT has been reported as 28% and as high as 100% in untreated patients [4].

Presentation of patients with RHThIT is variable from mild respiratory symptoms, as in our case, to cardiogenic shock and sudden death. 

TTE is usually sufficient for the diagnosis of RHThIT and considered as a screening test, with 50 to 60% sensitivity for detection of right heart thrombi but may underestimate the clot size whereas TEE not only detects the thrombus in the heart with higher accuracy it may also allow diagnosis of pulmonary embolism with 80% sensitivity and 100% specificity in patients with suspected massive pulmonary emboli [5, 6]. Echocardiography provides information for the presence of a right-to-left shunt through a patent foramen ovale (PFO). On the other hand, contrast-enhanced CT scanning (Helical CT) is also a cheap and noninvasive technique that will diagnose not only pulmonary embolism but also right atrial thrombus with high accuracy. However, CT could not be done in our case due to high creatinine. The combination of a right atrial clot and a PFO could lead to the paradoxical embolization of the arterial circulation. Thus, increasing the urgency for prompt treatment. Because the estimated prevalence of PFO in the general population (ranges between 10% to 35%) is high, echocardiographic examination became an important initial testing in patients with massive pulmonary embolism.

The optimal therapy for RHThIT is an ongoing debate because prospective randomized controlled studies are lacking. 

Existing published reports differ in their recommendations for treatment by advocating surgical removal, administration of thrombolytic agents,anticoagulation therapy with heparin, or using interventional percutaneous thrombus retrieval techniques. 

Kinney and Wright [7] found similar mortality rates for surgery, thrombolysis, and anticoagulation with heparin (38%, 38%, and 30%, resp.) and thus concluded that heparin therapy was the treatment of choice, because of its safety profile.

Similarly, no mortality benefit was found at 3 months with treatment of heparin, thrombolysis, or embolectomy (29%, 29%, and 25%) in the report of International Cooperative Pulmonary Embolism Registry [8].

In a retrospective analysis Rose et al. [4], 98% of analyzed 177 patients with right heart thromboembolism had pulmonary embolism. While 9% did not receive treatment, others received anticoagulation alone (35%), surgical thrombectomy (35.6%), or thrombolytic treatment (19.8%). The mortality rate associated with no therapy, anticoagulation, surgical embolectomy, and thrombolysis was 100%, 28.6%, 23.8%, and 11.3%, respectively. Subgroup-analysis, in the sample of 123 patients, who underwent surgical or thrombolytic therapy, surgery was associated with an increased risk of mortality (OR, 2.83, 95% CI, 1.04 to 7.69). Multivariate modeling for survival as the primary outcome, thrombolytic therapy was associated with an improved survival rate (*P* < 0.05), when compared to either anticoagulation therapy or surgery. 

On contrary, Chartier et al. reported a series of 38 patients with free-floating thrombi in the right heart that treated during a 12-year period. They found that the mortality rate was high regardless of the therapeutic option chosen: surgery (47.1%), thrombolytic agents (22.2%), heparin alone (62.5%), and interventional percutaneous technique (50%) [3].

Although thrombolysis is a simple and fast treatment option with numerous advantages including acceleration of pulmonary reperfusion, reduction in pulmonary hypertension, improvement of right ventricular function, possibility of dissolving the intracardiac thrombus, pulmonary embolism, and the venous thromboembolism at the same time, possibility of the clot breaking loose and embolization to the lungs where there is already thrombus and bleeding (major bleeding risk 22%, cerebral hemorrhage 3%) might be problematic [9].

A more recent prospective study found a good outcome and rapid improvement of echocardiographic and scintigraphic parameters after thrombolysis with rt-PA in 9 patients with mobile right heart thrombus and massive pulmonary embolism [10].

Mortality in patients with right heart thrombi remains high regardless the cause or the chosen treatment. 

Although there is no clear consensus for preferred treatment for RHThIT, rapid diagnosis and management is essential. As a general rule, an individualized approach based on characteristics of the patients and thrombus is needed. Factors that should be considered include extent, size, shape, mobility of the thrombus, preexisting pulmonary embolism, DVT and cardiopulmonary reserve of the patient. 

Nearly all patients with RHThIT have coexisting massive bilateral pulmonary emboli. Thus, anticoagulation treatment alone could be hazardous given the presence of a free-floating thrombus in the right heart that could embolize to a severely compromised pulmonary circulation.

Surgical thromboembolectomy with exploration of the right chambers is considered as the classical treatment for right heart thrombus. But, it has its own potential complications including general anesthesia, cardiopulmonary bypass, and inability to remove coexisting pulmonary thromboemboli beyond the central pulmonary arteries. Those with thrombus in one chamber (atrium or ventricle) or in the PFO, massive pulmonary embolism in central pulmonary arteries, and stable enough for surgery can be good candidates for surgical approach. 

Thrombolytic treatment may be an option when surgery is contraindicated and the patient is hemodynamically unstable but should be avoided in patients with thrombus that traverses at patent foramen ovale to the left atrium because thrombolysis may cause thrombus fragmentation and systemic embolization.

Catheter-based interventions might be options when surgery and lytic therapy are contraindicated. These percutaneous techniques include aspiration thrombectomy (the Greenfield suction embolectomy catheter), thrombus fragmentation (pig tail rotational catheter, Amplatzer catheter), and rheolytic thrombectomy (Hydrolyse, Angiojet and Oasis catheters). Although this modality is not readily available and there is no enough experience but it can be complimentary to surgery when the pulmonary arterial clots cannot be recovered surgically [11]. 

It is important to scan extremities for deep vein thrombosis once the massive pulmonary embolism is diagnosed. Retrievable IVC filters may be required preventing further clot traveling.

## 4. Conclusion

Right heart thrombi-in-transit and deep venous thrombus should be sought in patients with massive pulmonary embolism. Echocardiography is necessary to assess the presence of PFO since the therapeutic options may vary in patients with right heart thrombus.
